# Supplementary material for: Environmental Tobacco Smoke Exposure in Relation to Family Characteristics, Stressors and Chemical Co-Exposures in California Girls
Source: Int J Environ Res Public Health. 2019 Oct 30;16(21):4208. doi: 10.3390/ijerph16214208 (PMC6862570; doi:10.3390/ijerph16214208)
Supplement: Supplementary file 1 [file ijerph-16-04208-s001.pdf]

Supplementary Material for:

Environmental Tobacco Smoke Exposure in Relation to Family Characteristics, Stressors and Chemical Co-Exposures in California Girls

GC Windham et al.

Table S1: Additional Biomarkers of Exposure Measured among Participants

Table S2: Urinary Cotinine Geometric Mean (GM) Levels (μg/g creatinine) by Demographic and Household Characteristics among Girls in "Non-smoking" Households

Table S3: Urinary Cotinine Geometric Mean (GM) Levels (μg/g creatinine) by Stressful Life Events among Girls in "Non-smoking" Households

Table S1. Additional Biomarkers of Exposure[1] Measured among Participants

Analyte Category	Panel Compounds/Congeners
Urinary Metal (ug/g creatinine)	antimony, arsenic, barium, beryllium, cadmium, cesium, cobalt, lead, manganese, molybdenum, platinum, strontium, thallium, tin, tungsten, uranium
Blood Metal (ug/L)	arsenic, cadmium, lead, manganese, mercury
Urinary Polycyclic Aromatic Hydrocarbons (PAHs) (ng/g creatinine)	2-OH-fluorene (fluor-2), 3-OH-fluorene (fluor-3), 9-OH-fluorene (fluor-9), 1-napthol (nap-1), 2-napthol (nap-2), 1-OH-phenanthrene (phen-1), 2-OH-phenanthrene (phen-2), 3-OH-phenanthrene (phen-3), 4-OH-phenanthrene (phen-4), 1-hydroxypyrene (pyr_1)
Urinary Phthalate metabolites (ug/g creatinine)	Mono-n-butyl phthalate (mBP), Monobenzyl phthalate (mBzP2), Mono-3-carboxypropyl phthalate (mCPP), Mono-2-ethyl-5-carboxypentyl phthalate (mECPP), Mono-2-ethylhexyl phthalate (mEHP), Mono-2-ethyl-5-oxohexyl phthalate (mEOHP), Monoethyl phthalate (mEP2), Mono-isobutyl phthalate (miBP)
Urinary Phenol metabolites (ug/g creatinine)	butyl paraben (B-PB), bisphenol A, 2,4-dichlorophenol (24-DCP), 2,5-dichlorophenol (25-DCP), methyl paraben (M-PB), propyl paraben (P-PB), triclosan (TCS)
Serum Per- and Poly-Fluoroalkyl substances (PFASs) (ug/L)	2-(N-methyl-perfluorooctane sulfonamido) acetate (Me-PFOSA-AcOH), perfluorodecanoate (PFDeA), perfluorohexane sulfonate (PFHxS), perfluorononanoate (PFNA), perfluorooctane sulfonate (PFOA)
Serum Polybrominated Diphenyl Ethers (PBDEs) (ng/g Lipid)	PBDE-28, PBDE-47, PBDE-85, PBDE-99, PBDE-100, PBDE-153, PBDE-154
Serum Polychlorinated Biphenyls (PCBs) (ng/g Lipid)	PCB-74, PCB-99, PCB-105, PCB-118, PCB-138/158, PCB-146, PCB-153, PCB-156, PCB-180, PCB-187, PCB-196/203
Organo-Chlorinated Pesticides (OCPs) (ng/g Lipid)	hexachlorobenzene (HCB), oxychlordane (OXYCHLOR), p,p'-Dichlorodiphenyldichloroethylene (PP-DDE), trans-Nonachlor (T-NONA)

[1] Table includes all chemical classes/congeners in biomarker panels, even those not included in factor analysis due to <60% above limit of detection.

Table S2. Urinary Cotinine Geometric Mean (GM) Levels (µg/g creatinine) by Demographic and Household Characteristics among Girls in "Non-smoking" Households

Characteristics	N=341	Unadjusted			Adjusted[1]		
	%	GM	95% CI	p	GM[2]	95% CI	p
Race							
Asian	13	0.25	(.19-.34)	0.23	0.26	(.19-.34)	0.77
Black	20	0.33	(.25-.44)	<.01	0.26	(.21-.33)	0.60
Hispanic	23	0.26	(.22-.30)	0.12	0.22	(.18-.28)	0.54
N-H White	44	0.21	(.18-.24)	ref	0.24	(.21-.29)	ref
Family Income							
>=$100k/yr	45	0.20	(.18-.23)	ref	0.24	(.20-.28)	ref
>=$50, <100k/yr	37	0.26	(.22-.30)	.03	0.25	(.21-.29)	0.69
<$50k/yr	17	0.37	(.29-.47)	<.01	0.27	(.20-.36)	0.51
Primary Caregiver Education							
Bachelor or Higher	57	0.21	(.19-.24)	ref	0.23	(.20-.26)	ref
Some College	27	0.33	(.26-.41)	<.01	0.29	(.24-.35)	0.04
High School or less	16	0.28	(.22-.34)	.05	0.24	(.18-.32)	0.64
Home Ownership							
Owns Home	76	0.21	(.19-.24)	ref	0.22	(.20-.25)	ref
Is Renter	24	0.38	(.30-.47)	<.01	0.32	(.26-.40)	<.01
Two Parents in Household[3]							
Yes	84	0.22	(.20-.25)	ref	0.23	(.21-.26)	ref
No	16	0.42	(.31-.55)	<.01	0.32	(.24-.42)	0.05
Siblings in Household[4]							
Yes	80	0.24	(.22-.27)	ref	0.24	(.22-.27)	ref
No	20	0.27	(.21-.35)	0.33	0.26	(.20-.32)	0.68
Crowding (# ppl/ # rooms)							
Not Crowded (< 1)	92	0.24	(.21-.26)	ref	0.24	(.22-.26)	ref
Crowded (>=1)	8	0.40	(.24-.64)	<.01	0.33	(.23-.46)	0.10

[1] Adjusted for all listed independent variables (N=336). [2] Geometric means were calculated using least squares means. [3] Parent was considered a biological parent. [4] Sibling was considered a biological sibling.

Table S3. Urinary Cotinine Geometric Mean (GM) Levels (μg/g creatinine) by Stressful Life Events among Girls in "Non-smoking" Households.

Stressful Life Events	N=269	Unadjusted			Adjusted[1]		
	%	GM[2]	95% CI	p	GM[2]	95% CI	p
**Pregnancy**							
Total Number of Stressful Life Events							
None	68	0.25	(.22-.29)	ref	0.25	(.22-.29)	ref
1-2 events	29	0.24	(.20-.29)	0.72	0.23	(.19-.29)	0.50
3 or more events	2	0.25	(.06-.99)	0.99	0.24	(.12-.49)	0.89
Unemployment in the family							
No	93	0.25	(.22-.28)	ref	0.25	(.22-.28)	ref
Yes	7	0.24	(.14-.40)	0.79	0.22	(.14-.33)	0.50
Illness or injury in the family							
No	91	0.25	(.22-.28)	Ref	0.25	(.22-.28)	ref
Yes	9	0.22	(.15-.31)	0.46	0.22	(.15-.31)	0.45
Death of close family member							
No	91	0.25	(.22-.28)	Ref	0.25	(.22-.28)	ref
Yes	9	0.26	(.18-.37)	0.82	0.27	(.19-.38)	0.61
Separation or divorce							
No	90	0.24	(.22-.27)	ref	0.25	(.22-.28)	ref
Yes	10	0.31	(.21-.45)	0.21	0.27	(.19-.38)	0.56
Legal or financial problems							
No	93	0.25	(.23-.29)	ref	0.25	(.23-.28)	ref
Yes	7	0.18	(.12-.28)	0.15	0.18	(.12-.27)	0.09
**Childhood (0-6 years)**							
Total Number of Stressful Life Events							
None	36	0.23	(.20-.27)	ref	0.24	(.20-.29)	ref
1-2 events	44	0.25	(.21-.30)	0.55	0.25	(.21-.29)	0.78
3 or more events	19	0.28	(.22-.37)	0.23	0.26	(.20-.33)	0.64
Unemployment in the family							
No	79	0.23	(.20-.26)	ref	0.23	(.21-.26)	ref
Yes	21	0.34	(.25-.46)	<.01	0.32	(.25-.40)	0.02
Illness or injury in the family							
No	71	0.26	(.23-.30)	ref	0.26	(.23-.30)	ref
Yes	29	0.22	(.18-.28)	0.19	0.22	(.18-.27)	0.15
Death of close family member							
No	61	0.26	(.23-.31)	ref	0.26	(.22-.30)	ref
Yes	39	0.23	(.19-.27)	0.22	0.23	(.20-.28)	0.38
Separation or divorce							
No	78	0.23	(.21-.26)	ref	0.24	(.21-.27)	ref
Yes	22	0.32	(.24-.42)	0.02	0.28	(.22-.35)	0.30
Legal or financial problems							
No	83	0.25	(.22-.28)	ref	0.25	(.22-.28)	ref
Yes	17	0.27	(.20-.36)	0.59	0.23	(.18-.30)	0.62

[1] Adjusted for primary caregiver education (3-levels) and home ownership (yes/no). [2] Geometric means and 95% confidence intervals were calculated using least squares mean.
